# Our Confreres of the Japanese Embassy

**Published:** 1860-07

**Authors:** 


### OUR CONFRERES OF THE JAPANESE EMBASSY.

An interesting interview occurred a few days since between Dr. Hills, Superintendent of the Columbus (O.) Lunatic Asylum, and the physicians to the Japanese Embassy. The following colloquy took place:

Dr. Hill—How many insane persons have you in Japan? Very few.

Have you separate hospitals for them? We have four hospitals in Jeddo for the sick, with separate wards for the insane.

Do you use force or violence in their management? We do not, but have strong rooms and guards.

Do you ever bleed insane patients? Never.

Are idiots and lunatics kept in the same hospitals? They are, but in different wards; we have but few; not more than twenty in all; there may be some in private hospitals.

How many sick do you average in your hospitals? From five to eight hundred, but all poor.

Here the Japanese doctors became interrogators, and inquired:

Have you many insane?

Dr. Hill—We have three hundred in my hospital.

How many of these are insane? All.

This reply astonished the inquisitors, who raised their hands and looked at each other.

What medicines do you use? Wines, quinine, and other stimulants.

Have you hospitals for the dumb and blind? Yes, but separate.

Have you medicinal gardens? None of importance.

The Japanese here remarked that they would like to get the seed of our plants of every description, for the imperial gardens of Jeddo, and they were informed these would be furnished them by the National Agricultural Society. They were also told that they would have an opportunity to inspect the Asylum for the Insane before leaving Washington, which appeared to gratify them very much.

---

☞ Dr. Letheby, of London sanitary reputation, condemns the scheme which has been adopted of deodorizing the river Thames with per-chloride of iron.

